# Psychological readiness for national examinations: study habits, anxiety, and motivation among secondary school students in Somaliland

**DOI:** 10.3389/fpsyg.2026.1776290

**Published:** 2026-05-25

**Authors:** Abdirahman Ahmed Mohamoud, Mukhtaar Axmed Cumar, Jibril Abdikadir Ali

**Affiliations:** 1 School of Postgraduate Studies and Research, Amoud University, Hargeisa, Somalia; 2 Faculty of Education, Amoud University, Borama, Somalia; 3 College of Education and Behavioral Science, University of Hargeisa, Hargeisa, Somalia

**Keywords:** academic performance, anxiety, motivation, national examinations, psychological readiness, secondary school students, study habits

## Abstract

Psychological readiness plays a critical role in how secondary school students prepare for high-stakes national examinations. This study examined the relationships among study habits, examination anxiety, academic motivation, and perceived readiness among secondary school students in Somaliland. A cross-sectional predictive correlational design was employed with a sample of 539 Form 4 students. Data were collected using a structured self-report questionnaire administered online. Pearson correlation analysis showed that study habits were positively associated with academic motivation (*r* = 0.610, *p* < 0.001) and perceived readiness (*r* = 0.628, *p* < 0.001), while examination anxiety was negatively associated with both study habits (*r* = −0.608, *p* < 0.001), academic motivation (*r* = −0.535, *p* < 0.001), and perceived readiness (*r* = −0.656, *p* < 0.001). Multiple regression analysis indicated that examination anxiety significantly and negatively predicted perceived readiness (B = −0.400, *p* < 0.001), whereas academic motivation (B = 0.202, *p* < 0.001) and study habits (B = 0.143, *p* < 0.001) were significant positive predictors. Together, the predictors explained 54.1% of the variance in perceived readiness. The findings show that perceived readiness is positively associated with stronger study habits and academic motivation, but negatively associated with examination anxiety, underscoring the need for comprehensive school-based support during high-stakes examination periods.

## 1 Introduction

High-stakes national examinations continue to shape educational trajectories across secondary education systems. In many low- and middle-income contexts, these examinations function as decisive gateways to higher education and employment. As a result, student performance reflects not only academic knowledge but also students' psychological capacity to cope with sustained pressure and evaluative demands (Kassaw and Demareva, 2023; Onoshakpokaiye, 2024; Putwain et al., 2023). Recent research increasingly suggests that examination outcomes cannot be fully understood without attention to students' perceived psychological readiness for high-stakes assessment.

Psychological readiness for national examinations is better understood as students' perceived preparedness to cope with academic demands, emotional pressure, and performance expectations during high-stakes assessment, rather than a stable trait. It reflects students' self-appraisal of how prepared they feel to face the examination context (Ragusa et al., 2023; Shin et al., 2023). Within this framework, study habits, examination anxiety, and academic motivation consistently emerge as closely related psychological factors that may shape students' readiness. However, many studies continue to examine these variables separately, limiting understanding of how they jointly relate to and predict readiness during high-stakes assessment (Soares and Woods, 2020; Steare et al., 2023).

Study habits represent an important behavioral predictor of perceived readiness. Contemporary evidence shows that structured routines, time management, and self-monitoring behaviors are associated with stronger engagement and perceived preparedness for examinations (Hinds and Sánchez, 2022; Ragusa et al., 2023; Sankalaite et al., 2021). Yet recent studies caution that effective study behaviors alone do not guarantee readiness. Emotional strain and perceived pressure can undermine even well-established preparation routines (Li et al., 2022a; Shin et al., 2023).

Examination anxiety has therefore become a central concern in recent education research. Elevated anxiety levels are associated with impaired attention, avoidance behaviors, and reduced confidence during exam preparation (Hu et al., 2022; Kritikou and Giovazolias, 2022; Martinez-Yarza et al., 2023). Anxiety is increasingly understood as an important psychological factor associated with students' readiness, one that both shapes, and may be shaped by, students' study behaviors and motivational orientation (Putwain et al., 2023; Tareke et al., 2023). Despite this recognition, anxiety is often treated as a standalone risk factor rather than examined alongside other psychological predictors of readiness.

Motivation provides the regulatory force that links behavior and emotion. Recent studies suggest that motivated students persist longer, engage more deeply with learning tasks, and cope more effectively with academic pressure (Froiland, 2020; Iliescu and Greiff, 2022; Li F. et al., 2023; Li R. et al., 2023; Wagner, 2023). Motivation may also buffer the negative effects of anxiety by sustaining engagement when stress levels rise (Shin et al., 2023; Soares and Woods, 2020). However, the relative contribution of motivation to students' perceived readiness, compared with study habits and anxiety, remains underexamined in many secondary education studies.

These psychological dynamics are especially salient in fragile and low-resource education systems. Students in such contexts often face heightened examination pressure alongside limited institutional support and guidance services (Bond, 2023; Gakinya et al., 2022). Within Somaliland's secondary school system, Form 4 students sit for a high-stakes national examination at the end of secondary education, and performance in this examination plays an important role in shaping progression to further study and future opportunities. Yet empirical research focusing on students' psychological readiness for this examination context remains limited (Abbas et al., 2022; Kassaw and Demareva, 2023; Mohamed, 2022).

Globally, post-2020 scholarship increasingly calls for integrative models that examine study habits, anxiety, and motivation simultaneously rather than in isolation. Such approaches are necessary to identify dominant readiness factors and to inform targeted school-level interventions (Onoshakpokaiye, 2024; Ragusa et al., 2023; Steare et al., 2023). However, few studies have applied this approach in fragile or post-conflict secondary education systems, leaving a clear knowledge gap regarding how these psychological factors jointly shape readiness in such contexts.

This study responds to these gaps by examining how study habits, examination anxiety, and academic motivation relate to and predict students' perceived readiness for national examinations among secondary school students in Somaliland. Using correlational and regression-based analyses, the study investigates the joint and relative contribution of these psychological factors to perceived readiness. By focusing on psychological readiness rather than examination outcomes alone, the study aims to provide context-sensitive evidence to inform student support strategies in high-stakes assessment systems. In this study, perceived readiness is treated as a distinct outcome reflecting students' self-appraisal of their preparedness for the national examination context.

## 2 Literature review

### 2.1 Conceptualizing psychological readiness

Recent literature conceptualizes psychological readiness for examinations as students' perceived preparedness to cope with the academic, emotional, and performance demands of high-stakes assessment. Readiness reflects students' self-appraisal of how well prepared they feel to manage examination pressure, maintain engagement, and respond effectively to evaluative demands (Kassaw and Demareva, 2023; Onoshakpokaiye, 2024; Putwain et al., 2023). This framing moves beyond single-variable explanations and aligns with evidence showing that readiness is influenced by interacting psychological resources (Ragusa et al., 2023; Shin et al., 2023). Within this perspective, study habits represent one important behavioral factor associated with readiness. Recent studies show that planning, time management, and self-monitoring behaviors support engagement and preparedness, particularly during examination periods (Hinds and Sánchez, 2022; Sankalaite et al., 2021; Steare et al., 2023). However, these behaviors are not sufficient on their own. Their effectiveness depends on students' emotional regulation and motivational orientation (Chen and Wang, 2022; Li et al., 2022a; Ragusa et al., 2023).

Examination anxiety represents an important psychological factor associated with students' readiness for examinations. Contemporary research links elevated anxiety to impaired concentration, avoidance behaviors, and reduced perceived preparedness (Hu et al., 2022; Kritikou and Giovazolias, 2022; Martinez-Yarza et al., 2023; Sanusi et al., 2022; Shrestha and Das, 2022). Anxiety interacts with study behavior by discouraging persistence and increasing procrastination, particularly in high-stakes contexts (Putwain et al., 2023; Tareke et al., 2023; Tarekegne and Megersa, 2019). Motivation provides the regulatory mechanism connecting behavior and emotion. Recent studies show that motivated students are more likely to persist, adopt adaptive strategies, and maintain engagement under stress (Froiland, 2020; Ilie et al., 2021; Li R. et al., 2023; Wagner, 2023). Motivation therefore plays a central role in shaping students' perceived readiness for demanding examination contexts.

### 2.2 Theoretical perspectives

Contemporary theoretical work conceptualizes examinations as structured stress environments. Students' appraisal of examination demands shapes emotional responses and learning behaviors (Hu et al., 2022; Putwain et al., 2023; Tareke et al., 2023; Tarekegne and Megersa, 2019). These perspectives emphasize how pressure influences both cognition and behavior. Self-regulatory models further suggest that students' perceived readiness depends partly on their ability to align goals, effort, and coping strategies under pressure (Bond, 2023; Breslin and Bailey, 2020; Wagner, 2023). Within this view, motivation sustains engagement, while emotional regulation supports effective study behavior. Despite these advances, theoretical integration remains uneven. Many studies reference multiple frameworks without translating them into coherent analytic models that explain how study habits, examination anxiety, and academic motivation jointly relate to perceived readiness (Shin et al., 2023; Soares and Woods, 2020).

### 2.3 Empirical evidence

Empirical studies consistently demonstrate positive associations between study habits and readiness-related outcomes, including perceived preparedness and adaptive examination behavior (Ragusa et al., 2023; Sankalaite et al., 2021; Steare et al., 2023). Anxiety shows a robust negative association, particularly in examination-oriented systems (Kritikou and Giovazolias, 2022; Martinez-Yarza et al., 2023). Motivation often emerges as a dominant correlate when multiple psychological variables are examined simultaneously (Froiland, 2020; Ilie et al., 2021; Wagner, 2023). However, many studies rely on bivariate analyses, limiting insight into the joint and relative contribution of these factors to students' perceived readiness (Li et al., 2022a,b; Shin et al., 2023). Evidence from fragile and low-resource contexts remains limited. Existing studies suggest that institutional constraints amplify psychological pressure and increase reliance on individual coping mechanisms (Abdi et al., 2025; Bond, 2023; Gakinya et al., 2022).

### 2.4 Methodological gaps

Recent methodological reviews highlight persistent limitations in readiness research. These include limited construct integration and insufficient attention to context-specific measurement (Weinan et al., 2020; Iliescu and Greiff, 2022; Knottnerus and Tugwell, 2020). Many studies apply instruments without adaptation to examination systems in fragile contexts (Ali et al., 2025b; Saccone et al., 2020). Overall, the literature points to a clear methodological and knowledge gap. There is a need for integrative, context-sensitive models that examine study habits, anxiety, and motivation simultaneously to identify their joint and relative contribution to students' perceived readiness, particularly in fragile and examination-oriented contexts.

## 3 Methodology

### 3.1 Research design

This study employed a quantitative, non-experimental research approach using a cross-sectional predictive correlational design. The design was appropriate because the study sought to capture a snapshot of students' psychological conditions during the critical pre-examination period and to examine the relationships among the study variables at a single point in time. The correlational component was used to determine the direction and strength of the relationships among study habits, examination anxiety, academic motivation, and perceived readiness, while the predictive component was used to assess the extent to which study habits, examination anxiety, and academic motivation predicted students' perceived readiness for the Somaliland National Examinations. In this study, perceived readiness was treated as the dependent outcome variable, whereas study habits, examination anxiety, and academic motivation were treated as predictor variables. This distinction was maintained to ensure consistency between the conceptual framework, measurement approach, and statistical analysis.

### 3.2 Study area and population

The study was conducted in Somaliland and targeted secondary education institutions. The target population comprised Form 4 students enrolled in secondary schools and preparing for the Somaliland National Examinations. This population was selected because Form 4 represents the final grade of secondary education in Somaliland, and students at this level sit for a high-stakes national examination that plays an important role in determining progression to further education and future academic opportunities. As a result, these students are likely to experience substantial academic pressure, making them an appropriate population for examining psychological readiness in relation to examination preparation. The study included students from both public and private secondary schools in order to capture variation in educational context and access to academic support.

### 3.3 Sample size and sampling technique

The sample size was determined using Yamane's (1967) formula for finite populations at a 95% confidence level and a 5% margin of error (e = 0.05). Based on this procedure, a target sample size of 540 students was established. The final analyzed sample consisted of 539 respondents after data screening and cleaning.

The study adopted a stratified sampling approach at the planning stage in order to enhance representation across key subgroups of the target population. The main stratification criteria were region, gender, and school type. However, because the questionnaire was administered online through school communication channels, the final sampling process did not involve a strictly controlled probability-based random selection of individual students. The sample is therefore more accurately described as a cross-sectional online survey with stratified outreach efforts rather than a fully implemented stratified random sample.

The stratification considerations included region, gender, and school type. Participants were drawn primarily from Maroodi Jeex and surrounding areas, with efforts made to include both male and female students as well as students from government and private schools. Although the study sought to capture heterogeneity across these strata, participation was uneven across regions, with most respondents coming from Maroodi Jeex. This pattern may reflect both the concentration of students in that region and differences in accessibility to the online survey. Accordingly, the findings should be interpreted with caution in relation to regional representativeness. In practical terms, eligible students were reached through participating schools, and the questionnaire link was shared through official school WhatsApp groups and class representatives. Students who met the inclusion criteria and consented to participate completed the questionnaire voluntarily.

### 3.4 Instrumentation

Data were collected using a self-administered structured questionnaire titled the Psychological Readiness for Exams Scale (PRES). The instrument was hosted on Google Forms to facilitate remote data collection. The questionnaire was designed as a brief self-report tool to capture key indicators of students' preparation for the national examination. Rather than functioning as a full psychometric inventory, the tool provided questionnaire-derived composite measures aligned with the study objectives.

The questionnaire consisted of five sections. Section A collected demographic information, including gender, school type, and region. The remaining sections contained 10 self-assessment items related to students' examination preparation and emotional responses. For analysis, these items were organized into four questionnaire-derived composite measures. Study habits were derived from items assessing consistency of study routine, revision of past papers, time management, and sleep pattern before examinations (Q2, Q6, Q7, and Q10). Examination anxiety was derived from items assessing stress and pressure related to the examination (Q4 and Q8). Academic motivation was derived from items assessing persistence in difficult subjects and support-seeking behavior (Q3 and Q9). Perceived readiness was derived from items assessing students' feelings about the upcoming examination and their perceived performance in mock or practice examinations (Q1 and Q5).

All substantive items were measured using ordered response categories ranging from more positive to less positive states of readiness. During coding, responses were converted into numeric scores such that higher values reflected more favorable conditions on the original item. Where necessary, items used in the anxiety composite were reverse-coded during analysis so that the final examination anxiety score represented higher levels of anxiety. This approach allowed the composites to align with the final regression model in which study habits, examination anxiety, and academic motivation served as predictors of perceived readiness.

To ensure cultural and linguistic appropriateness, the instrument was translated from English into Somali. Conceptual equivalence was checked to ensure that key psychological terms retained their intended meaning in the local context. The translated version was also reviewed for clarity, contextual suitability, and comprehensibility before final administration. For measurement transparency, the full wording of the 10 questionnaire items used to derive the four composite measures is provided in Appendix A.

### 3.5 Validity and reliability

Content validity was established through an expert review process involving faculty members from the Department of General Psychology at Amoud University. The reviewers examined the items for clarity, relevance, ambiguity, and suitability to the Somaliland secondary school examination context. Based on their feedback, minor revisions were made to improve wording, clarity, and contextual appropriateness before the instrument was finalized for pilot testing and administration.

A pilot study was conducted with 30 Form 4 students who were not included in the final sample. Internal consistency was initially examined using Cronbach's alpha coefficient. In the final analysis, the questionnaire-derived composites demonstrated moderate to modest internal consistency. The Cronbach's alpha coefficients were 0.683 for study habits, 0.618 for examination anxiety, 0.610 for academic motivation, and 0.692 for perceived readiness. These coefficients suggest that the composites were sufficiently consistent for use as brief self-report indicators, although they were not intended to function as long-form psychometric scales. Accordingly, the findings should be interpreted with appropriate caution.

### 3.6 Data collection procedure

Formal permission to conduct the study was obtained from the relevant school administrations before data collection commenced. Because of logistical constraints and the need to reach students efficiently during the pre-examination period, the questionnaire was administered online using Google Forms. The survey link was distributed through official school WhatsApp groups and class representatives. A standard instruction set accompanied the link, explaining the purpose of the study and providing guidance on how to complete the questionnaire. Data collection took place over a 2-week period prior to the commencement of the national examinations.

Before accessing the questionnaire items, participants were presented with an introductory consent page explaining the purpose of the study, the voluntary nature of participation, the expected time required to complete the questionnaire, and the measures in place to protect anonymity and confidentiality. Only students who indicated their agreement were allowed to proceed to the questionnaire. In this way, informed consent was obtained electronically before participation. To improve response quality, participants were asked to complete the questionnaire independently and honestly. No personally identifying information was collected.

### 3.7 Data analysis

Data were cleaned and coded using Microsoft Excel before being exported to SPSS version 27 and Stata version 17 for analysis. Preliminary data screening was conducted to check completeness, coding accuracy, and consistency of responses before the main statistical analyses were performed.

The analysis was conducted in three stages. First, descriptive statistics, including frequencies, percentages, means, and standard deviations, were used to summarize demographic characteristics and the four main composite variables: study habits, examination anxiety, academic motivation, and perceived readiness. Second, Pearson's product-moment correlation coefficient was used to examine the strength and direction of the relationships among these four variables. Third, multiple linear regression analysis was conducted to assess the predictive power of study habits, examination anxiety, and academic motivation on perceived readiness. Multiple regression was appropriate because it allowed the estimation of the unique contribution of each predictor while controlling for the effects of the others.

Before conducting the regression analysis, key assumptions were examined, including normality, linearity, homoscedasticity, and multicollinearity. Diagnostic testing indicated no serious multicollinearity among the predictors, but some deviation from homoscedasticity and normality of residuals was observed. Therefore, the final regression model was estimated using **robust standard errors** to improve the reliability of inference. A significance level of *p* < 0.05 was adopted for all statistical tests.

### 3.8 Ethical considerations

Ethical approval for the study was obtained from the Research Ethics Review Committee of Amoud University. In addition, administrative permission to access schools and participants was obtained from the Regional Education Office and the administrations of the participating schools. Participation was voluntary, and informed consent was obtained electronically before data collection through the introductory page of the online questionnaire. Participants were informed of the purpose of the study, their right to decline participation or withdraw at any stage, and the confidential handling of their responses. Anonymity and confidentiality were strictly maintained throughout the study, and no personally identifying information was collected.

## 4 Results

### 4.1 Demographic characteristics of respondents

A total of 539 Form 4 secondary school students participated in the study. Table 1 presents the demographic characteristics of the respondents by sex and region. Female students constituted the majority of the sample (62.5%, *n* = 337), while male students accounted for 37.5% (*n* = 202). In terms of regional distribution, most respondents were drawn from Maroodi Jeex (94.6%, *n* = 510). The remaining participants were from Togdheer (2.2%, *n* = 12), Sahil (1.7%, *n* = 9), Awdal (0.6%, *n* = 3), Gabiley (0.6%, *n* = 3), and Sanaag (0.4%, *n* = 2). These figures indicate that the sample was heavily concentrated in Maroodi Jeex, with more limited representation from other regions.

**Table 1:** Demographic characteristics of respondents (*N* = 539).

Variable	Category	*n*	%
Sex	Male	202	37.5
	Female	337	62.5
Region	Maroodi Jeex	510	94.6
	Togdheer	12	2.2
	Sahil	9	1.7
	Awdal	3	0.6
	Gabiley	3	0.6
	Sanaag	2	0.4

### 4.2 Descriptive statistics of the main study variables

Descriptive statistics were computed for the four main composite variables included in the study: study habits, examination anxiety, academic motivation, and perceived readiness. As shown in Table 2, the mean score for study habits was 12.24 (SD = 2.59), indicating a moderate to relatively high level of positive study behavior among the respondents. The mean score for examination anxiety was 3.38 (SD = 1.32), suggesting that students reported varying but generally moderate levels of exam-related anxiety.

**Table 2:** Descriptive statistics of the main study variables (*N* = 539).

Variable	Mean	SD	Min	Max
Study habits	12.24	2.59	4	16
Examination anxiety	3.38	1.32	2	8
Academic motivation	6.29	1.50	2	8
Perceived readiness	6.12	1.39	2	8

The mean score for academic motivation was 6.29 (SD = 1.50), indicating that many students reported moderate to high levels of motivation and support-seeking behavior during exam preparation. The mean score for perceived readiness was 6.12 (SD = 1.39), suggesting that, on average, respondents perceived themselves as moderately prepared for the national examination. Overall, the descriptive results indicate meaningful variation across the four study variables, which justified proceeding to correlation and regression analyses.

### 4.3 Bivariate relationships among the main study variables

Pearson's product-moment correlation analysis was conducted to examine the relationships among study habits, examination anxiety, academic motivation, and perceived readiness. As presented in Table 3, study habits were positively and significantly correlated with perceived readiness (r = 0.628, *p* < 0.001), indicating that students with stronger study routines tended to report higher levels of readiness for the national examination. Similarly, academic motivation showed a positive and significant relationship with perceived readiness (r = 0.581, *p* < 0.001), suggesting that more motivated students were more likely to feel prepared for the examination.

**Table 3:** Pearson correlation matrix of the main study variables (*N* = 539).

Variable	1	2	3	4
1. Study habits	1.000			
2. Examination anxiety	−0.608^***^	1.000		
3. Academic motivation	0.610^***^	−0.535^***^	1.000	
4. Perceived readiness	0.628^***^	−0.656^***^	0.581^***^	1.000

^***^p < 0.001.

In contrast, examination anxiety was negatively and significantly associated with perceived readiness (*r* = −0.656, *p* < 0.001), indicating that higher levels of anxiety were linked to lower self-perceived readiness. In addition, study habits were negatively correlated with examination anxiety (*r* = −0.608, *p* < 0.001) and positively correlated with academic motivation (r = 0.610, *p* < 0.001). Examination anxiety also had a significant negative relationship with academic motivation (*r* = −0.535, *p* < 0.001). Overall, the correlation results suggest that positive study behavior and stronger motivation were associated with higher readiness, whereas examination anxiety was associated with reduced readiness.

### 4.4 Multiple regression analysis predicting perceived readiness

A multiple linear regression analysis with robust standard errors was conducted to determine the extent to which study habits, examination anxiety, and academic motivation predicted students' perceived readiness for the national examination. As shown in Table 4, the overall regression model was statistically significant, F_(3, 535)_ = 209.58, *p* < 0.001, and explained 54.1% of the variance in perceived readiness (*R*^2^ =0.5408). This indicates that the three psychological variables jointly made a substantial contribution to explaining differences in students' self-reported readiness.

**Table 4:** Multiple regression analysis predicting perceived readiness (*N* = 539).

Predictor	B	Robust SE	*t*	*p*	95% CI
Study habits	0.143	0.025	5.82	< 0.001	(0.095, 0.191)
Examination anxiety	−0.400	0.048	−8.34	< 0.001	(−0.495, −0.306)
Academic motivation	0.202	0.035	5.74	< 0.001	(0.133, 0.271)
Constant	4.452	0.417	10.69	< 0.001	(3.634, 5.270)

*R*^2^ = 0.5408; Model F_(3, 535)_ = 209.58, *p* < 0.001.

Among the predictors, academic motivation was a significant positive predictor of perceived readiness (B = 0.202, *p* < 0.001), indicating that students with higher levels of motivation and support-seeking behavior tended to report greater readiness. Study habits also showed a significant positive effect (B = 0.143, *p* < 0.001), suggesting that more consistent study routines, revision, time management, and healthier sleep patterns were associated with increased readiness. In contrast, examination anxiety was a significant negative predictor (B = −0.400, *p* < 0.001), meaning that higher anxiety levels were associated with lower perceived readiness for the national examination. Overall, the regression results show that readiness was positively shaped by study habits and academic motivation, while examination anxiety reduced students' sense of preparedness.

### 4.5 Assumption testing for the regression model

Diagnostic tests were conducted to assess whether the data met the assumptions required for multiple linear regression. Multicollinearity was examined using the variance inflation factor (VIF), and the results indicated no serious multicollinearity among the predictors. The VIF values were 1.94 for study habits, 1.70 for examination anxiety, and 1.71 for academic motivation, with a mean VIF of 1.78. These values were well below commonly accepted thresholds, suggesting that the predictor variables were sufficiently distinct for inclusion in the same regression model.

Additional diagnostic checks indicated some deviation from the assumptions of homoscedasticity and normality of residuals. The earlier Breusch–Pagan/Cook–Weisberg test suggested heteroskedasticity, and the Shapiro–Wilk test indicated that the residuals were not perfectly normally distributed. To address this issue and improve the robustness of the inferential results, the final regression model was estimated using robust standard errors. Visual inspection of the histogram, Q–Q plot, and residual-vs.-fitted plot further supported the decision to apply a robust estimation approach. Overall, the assumption checks suggested that while the data did not fully satisfy all classical regression assumptions, the final model remained appropriate for interpretation after correction for heteroskedasticity.

### 4.6 Reliability of the composite measures

The internal consistency of the composite measures was assessed using Cronbach's alpha coefficient. The results indicated moderate reliability across the four questionnaire-derived composites. As shown in Table 5, the study habits composite yielded an alpha coefficient of 0.683, while the examination anxiety composite produced an alpha of 0.618. The academic motivation composite had an alpha coefficient of 0.610, and the perceived readiness composite yielded an alpha of 0.692.

**Table 5:** Cronbach's alpha coefficients for the composite measures.

Composite measure	Number of items	Cronbach's alpha
Study habits	4	0.683
Examination anxiety	2	0.618
Academic motivation	2	0.610
Perceived readiness	2	0.692

Overall, these coefficients suggest that the measures demonstrated acceptable but modest internal consistency for a brief self-report instrument. The reliability values were somewhat lower than those typically expected for longer psychometric scales, which may reflect the short number of items in some of the composites, particularly the two-item measures. Therefore, the results should be interpreted with appropriate caution, while still providing useful preliminary evidence regarding the consistency of the questionnaire-derived indicators used in the study.

## 5 Discussion

This study examined how study habits, examination anxiety, and academic motivation were associated with and predicted secondary school students' perceived readiness for the Somaliland National Examinations. The findings showed that all three variables made significant contributions to perceived readiness. Study habits and academic motivation were positively associated with readiness, whereas examination anxiety was negatively associated with it. When considered together in the regression model, these factors explained a substantial proportion of the variation in students' perceived readiness. These results reinforce the view that students' preparation for high-stakes examinations is shaped not only by academic activity itself but also by how effectively they regulate behavior, manage stress, and sustain engagement under pressure (Kassaw and Demareva, 2023; Onoshakpokaiye, 2024; Putwain et al., 2023; Ragusa et al., 2023; Shin et al., 2023).

Academic motivation emerged as an important positive predictor of perceived readiness. This suggests that students who remain engaged, persistent, and willing to invest effort in their learning are more likely to feel prepared for demanding examinations. This finding is consistent with research showing that motivation supports sustained task involvement, adaptive coping, and continued effort in academically stressful situations (Froiland, 2020; Iliescu and Greiff, 2022; Wagner, 2023; Zhao and Li, 2023). Motivation may also help students maintain direction and confidence even when external pressure is high. In this sense, motivation does not merely accompany readiness; it appears to strengthen students' sense of preparedness by supporting persistence and purposeful engagement. This interpretation also aligns with studies suggesting that motivated learners are better positioned to manage setbacks and remain academically buoyant during periods of heightened assessment pressure (Putwain et al., 2023; Soares and Woods, 2020).

Study habits were also positively associated with perceived readiness, indicating that students with more consistent study routines, better time management, and more structured preparation tended to report higher levels of readiness. This finding supports previous evidence that organized study behavior contributes to preparedness, engagement, and effective performance-related adjustment (Hinds and Sánchez, 2022; Ragusa et al., 2023; Sankalaite et al., 2021; Steare et al., 2023). At the same time, the discussion should not overstate the role of study habits in isolation. The present results suggest that positive study behavior matters, but its contribution operates alongside emotional and motivational conditions. Students may have routines and plans, yet still feel insufficiently prepared if anxiety is high or motivation is weak. This interpretation is in line with research showing that behavioral strategies are most effective when accompanied by emotional regulation and adaptive self-management (Li, 2022; Li and Guo, 2022; Shin et al., 2023; Sun and Li, 2022; Tan and Liu, 2022). The findings therefore support a more integrated understanding of exam preparation in which behavioral discipline is important but not independently sufficient.

Examination anxiety showed a strong negative association with perceived readiness and remained a significant negative predictor in the multivariable model. This indicates that students who reported higher levels of anxiety were less likely to feel ready for the national examination. Such a pattern is consistent with literature linking examination anxiety to impaired concentration, avoidance behavior, uncertainty, and reduced confidence during preparation (Kritikou and Giovazolias, 2022; Martinez-Yarza et al., 2023; Salas-Pilco et al., 2022). Anxiety may interfere with readiness by disrupting students' ability to focus, organize effort, and interpret their own preparation positively. It may also weaken the benefits of otherwise effective study routines by increasing emotional strain and cognitive overload. This helps explain why anxiety continued to matter even after accounting for study habits and motivation. Related research similarly suggests that examination stress can influence not only how students feel, but also how they engage with learning tasks and manage academic setbacks (Putwain et al., 2023; Tareke et al., 2023; Tarekegne and Megersa, 2019). In the present study, anxiety appears to function as a meaningful barrier to perceived preparedness in a high-stakes assessment environment.

The Somaliland context gives these findings additional significance. In fragile and low-resource educational settings, students often prepare for examinations under conditions of constrained institutional support, limited guidance services, and uneven access to learning resources. Under such circumstances, psychological factors may become even more influential in shaping how students experience examination preparation (Bond, 2023; Gakinya et al., 2022). The strong concentration of examination pressure within a single high-stakes system means that students' perceived readiness may depend not only on academic competence, but also on the coping resources they can mobilize individually. Recent work from Somaliland also points to the broader influence of structural and contextual inequalities on academic experiences and outcomes (Ali et al., 2025a; Idris et al., 2025). For this reason, the present findings should be understood not simply as individual psychological patterns, but as patterns unfolding within a demanding examination environment where support systems may not always be sufficient.

These findings have practical implications. They suggest that efforts to improve examination preparedness should not focus only on study skills or content coverage. Interventions that target study routines alone may have limited effect if students remain highly anxious or poorly motivated. A more effective approach is likely to be one that addresses behavioral preparation, motivation enhancement, and anxiety management together. School-based guidance, teacher encouragement, structured revision support, and basic psychosocial strategies may all contribute to stronger readiness in an integrated way (Jörns-Presentati et al., 2021; Wennerhold and Friese, 2023). At the same time, the findings should be interpreted cautiously. The cross-sectional design does not permit causal conclusions, and the associations observed here are likely to be reciprocal and dynamic rather than strictly one-directional (Gallardo-Pujol et al., 2022; Kinnunen et al., 2023; Lee et al., 2023; Mills et al., 2021). Nevertheless, the results provide useful evidence that perceived readiness for high-stakes national examinations is meaningfully associated with students' study habits, anxiety, and academic motivation, and that examining these factors together offers a more realistic picture of students' examination preparation than considering any single factor in isolation.

## 6 Conclusion

This study examined perceived psychological readiness for national examinations among Form 4 secondary school students in Somaliland by focusing on three key psychological factors: study habits, examination anxiety, and academic motivation. The findings showed that perceived readiness was not associated with a single factor alone. Rather, it was shaped by a combination of behavioral, emotional, and motivational influences. Students with stronger study habits and higher academic motivation tended to report greater readiness for the national examination, whereas those with higher examination anxiety tended to report lower readiness. Taken together, these findings suggest that readiness for high-stakes assessment is closely connected to how students prepare, regulate stress, and sustain engagement during the examination period. The study makes an important contribution by shifting attention from examination outcomes alone to students' perceived preparedness before the examination takes place. This is particularly valuable in contexts such as Somaliland, where national examinations carry major consequences for educational progression and where institutional support systems may be limited. By examining study habits, anxiety, and motivation together, the study provides a more realistic picture of the psychological conditions under which students prepare for high-stakes examinations. It also responds to an important gap in the literature, where these factors are often examined separately rather than jointly.

The findings should be interpreted with appropriate caution. The study does not claim that these psychological factors cause readiness in a simple linear way. Rather, it shows that meaningful associations exist and that these associations matter for understanding students' examination experiences. Within this sample, examination anxiety emerged as a clear barrier to readiness, while motivation and study habits functioned as supportive factors. This pattern highlights the value of integrated student support approaches that address emotional well-being, motivation, and effective preparation simultaneously. Overall, the study shows that perceived readiness is a meaningful educational outcome and that strengthening students' readiness requires attention not only to academic content, but also to the psychological conditions that shape how students approach demanding examinations.

### 6.1 Implications for policy and practice

The findings of this study have important implications for both education policy and school-level practice. First, they suggest that examination readiness should be treated as more than an academic issue. In high-stakes systems, students' performance is shaped not only by curriculum coverage and subject knowledge, but also by the psychological conditions under which they prepare for examinations. This means that schools and education authorities should pay greater attention to students' readiness before the examination period, not only to results after the examination has taken place. At the school level, the results indicate that interventions should be integrated rather than narrowly academic. Programs that focus only on revision techniques or time management may be useful, but they are unlikely to be sufficient if students are also struggling with high anxiety or weak academic motivation. Schools should therefore consider practical support strategies that combine study-skills guidance with encouragement, emotional support, and simple stress-management approaches. For example, teachers can help students build structured revision plans, normalize examination-related stress, reinforce persistence, and identify students who appear overwhelmed or disengaged. These are relatively low-cost actions that can be implemented even in resource-constrained contexts.

The results also have implications for guidance and counseling provision. Examination anxiety emerged as a significant negative predictor of readiness, which suggests that anxiety should not be treated as a minor personal issue or as an individual weakness. Instead, it should be recognized as an educational concern that can interfere with students' ability to feel prepared and perform effectively. Where formal counseling services are limited, schools can still provide basic psychosocial support through trained teachers, school leaders, and peer-support mechanisms. Brief orientation sessions before examinations, reassurance about expectations, and opportunities for students to discuss challenges may help reduce unnecessary pressure. At the policy level, education authorities may consider incorporating psychological readiness into broader student-support frameworks. Monitoring indicators such as study habits, motivation, and examination anxiety could help schools identify students who may need additional support before high-stakes examinations. Teacher professional development is also important. Teachers are often the closest adults to students during examination preparation, and training them to recognize signs of stress, disengagement, or poor preparation could strengthen early support. More broadly, the findings suggest that educational quality should be defined in a wider sense. Strong systems do not only prepare students academically; they also help students cope, persist, and approach assessment with confidence and realistic readiness.

### 6.2 Study limitations

Several limitations should be considered when interpreting the findings of this study. First, the study used a cross-sectional design, which means that all variables were measured at a single point in time. As a result, the findings show association rather than causation, and the direction of influence among study habits, anxiety, motivation, and perceived readiness cannot be established with certainty. Second, the study relied on self-reported data collected through an online questionnaire. Such data may be affected by social desirability, misunderstanding of items, or differences in how students evaluate their own readiness and emotional experiences. Third, the sample was heavily concentrated in Maroodi Jeex, with much smaller representation from other regions. This limits the extent to which the findings can be generalized to all Form 4 students across Somaliland. Fourth, the composite measures used in the study were brief questionnaire-derived indicators rather than full psychometric scales, and some of the reliability coefficients were modest. Although they were adequate for the purposes of this exploratory analysis, they suggest that the measures should be interpreted with caution. Finally, the study focused on a limited set of psychological predictors. Other relevant influences, such as family support, school climate, teacher expectations, peer relationships, and socioeconomic conditions, were not included in the model and may also contribute to students' perceived readiness.

### 6.3 Recommendations and future research

Several recommendations follow from the findings of this study. At the practice level, schools should adopt more integrated approaches to examination preparation. Rather than treating revision, motivation, and emotional well-being as separate concerns, schools should address them together. Students may benefit from structured revision support, encouragement to persist in difficult subjects, and simple school-based strategies that help reduce excessive examination stress. Teachers and school leaders should be encouraged to recognize that students' sense of readiness is shaped not only by how much they study, but also by how motivated and emotionally supported they feel during the examination period. At the system level, education authorities should consider strengthening school-based support mechanisms before national examinations. This may include short preparation programs, teacher orientation on examination-related stress, and the use of simple screening indicators to identify students who appear poorly prepared, highly anxious, or disengaged. Because formal counseling services may be limited in many schools, future policy efforts should focus on practical and scalable support models that fit the realities of low-resource settings.

For future research, longitudinal studies are strongly recommended. Following students over time would help clarify how study habits, anxiety, motivation, and perceived readiness develop across the examination cycle. Such designs would also provide stronger evidence about temporal ordering and possible reciprocal relationships among the variables. In addition, future studies should broaden the range of predictors examined. Variables such as parental support, school climate, access to learning resources, previous academic performance, and teacher feedback may provide a fuller understanding of readiness in high-stakes contexts. Measurement development is another important area for future work. The present study used brief questionnaire-derived composites, which were useful for an initial investigation but should be refined in later studies. Future researchers should consider developing and validating more robust, context-sensitive instruments for assessing students' perceived readiness for national examinations in Somaliland and similar settings. Comparative studies across regions and school types would also be valuable, particularly given the uneven regional participation in the present sample. Finally, intervention-based research is needed. Experimental or quasi-experimental studies could test whether integrated support programs focused on study behavior, anxiety management, and motivation enhancement improve students' readiness and, eventually, their examination experiences and outcomes.

## Data Availability

The raw data supporting the conclusions of this article will be made available by the authors, without undue reservation.

## Appendix

**Appendix A**. Full Wording of the Questionnaire Items Used in the Composite Measures

The questionnaire included the following 10 self-assessment items, which were used to derive the composite measures of study habits, examination anxiety, academic motivation, and perceived readiness.

**Perceived readiness**

1. How do you feel about the upcoming national exam?

(A) Confident—I have prepared well.
(B) A bit nervous, but I believe I can do well.
(C) Very anxious—I'm not sure if I'm ready.
(D) Scared—I feel unprepared and overwhelmed.

2. How do you perform in past mock exams or practice tests?

(A) I do well and feel confident.
(B) I perform okay but know I need improvement.
(C) I struggle with some subjects and worry about my results.
(D) I perform poorly and feel discouraged.

**Study Habits**

2. How consistent has your study routine been?
(A) Very consistent—I study regularly and follow a plan.
(B) Fairly consistent—I study, but sometimes I get distracted.
(C) Inconsistent—I only study when exams are near.
(D) I barely study—I rely on last-minute revision.

6. How often do you revise and review past papers?

(A) Regularly—I solve past papers as part of my study plan.
(B) Sometimes—I review past papers when I have time.
(C) Rarely—I only look at them before the exam.
(D) Never—I don't use past papers for revision.

7. What is your approach to time management during exams?

(A) I practice answering questions within the time limit.
(B) I try to manage my time but sometimes struggle.
(C) I often run out of time in exams.
(B) I don't think about time management—I just answer as I go.

8. What is your sleeping pattern before exams?

(A) I maintain a healthy sleep routine.
(B) I sometimes sacrifice sleep for studying.
(C) I sleep very little before exams due to stress.
(D) I barely sleep, and I feel exhausted during exams.

**Examination Anxiety**

4. How do you deal with exam pressure and stress?

(A) I stay calm and use stress-management techniques.
(B) I get nervous but try to stay positive.
(C) I feel overwhelmed and stressed most of the time.
(D) I panic and struggle to concentrate.

8. How do you feel about the importance of the national exam?
(A) It's an important milestone, but I trust my preparation.
(B) It's important, but I try not to stress too much.
(C) It feels overwhelming, and I worry a lot about failing.
(D) I see it as a life-or-death situation, and it stresses me.

**Academic Motivation**

3. How do you handle difficult subjects or topics?

(A) I put extra effort into them and seek help.
(B) I try my best but sometimes avoid them.
(C) I struggle and often leave them until the last minute.
(D) I completely avoid them and hope for the best.

9. Do you seek help when you don't understand a topic?
(A) Always—I ask teachers, classmates, or tutors.
(B) Sometimes, but I try to figure it out alone first.
(C) Rarely—I feel embarrassed to ask for help.
(D) Never—I just move on and hope it doesn't appear in the exam.
